# 3,3′-Bis(3,4,5-trimethoxy­benzo­yl)-1,1′-(*o*-phenyl­ene)dithio­urea ethanol solvate

**DOI:** 10.1107/S1600536808023556

**Published:** 2008-07-31

**Authors:** Hai-Tang Du, Hai-Jun Du

**Affiliations:** a Institute of Natural Products, Research Center for Eco-Environmental Sciences, Guiyang College, Guiyang 550005, People’s Republic of China; b School of Chemistry and Environmental Science, Guizhou University for Nationalities, Guiyang 550025, People’s Republic of China

## Abstract

In the mol­ecule of the title compound, C_28_H_30_N_4_O_8_S_2_·C_2_H_6_O, the benzene ring is oriented at dihedral angles of 38.50 (6) and 5.68 (5)° with respect to the trimethoxy­phenyl rings, while the two trimethoxy­phenyl rings are oriented at a dihedral angle of 44.18 (5)°. Intra­molecular N—H⋯O and N—H⋯S hydrogen bonds result in the formation of non-planar six-, seven- and eight-membered rings. The twisting modes of the two side arms are different [C—N—C—O and C—N—C—N torsion angles = 0.1 (3) and 11.8 (3)°, respectively, in one arm, and 4.6 (3) and −11.5 (3)° in the other]. In the crystal structure, inter­molecular N—H⋯O and O—H⋯O hydrogen bonds link the mol­ecules.

## Related literature

For a related structure, see: Thiam *et al.* (2008 ▶). For ring conformation puckering parameters, see: Cremer & Pople (1975 ▶).
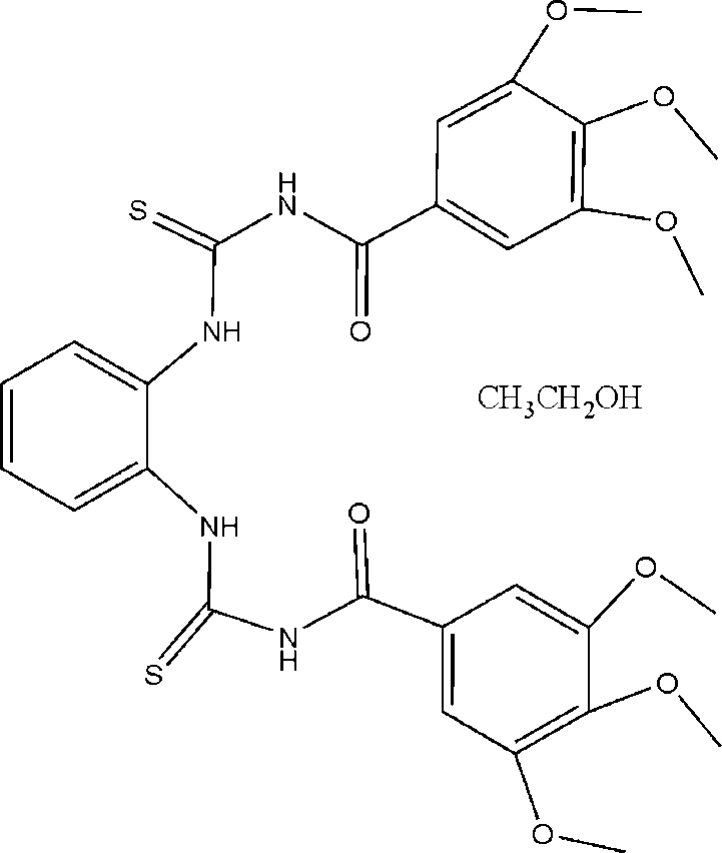


## Experimental

### 

#### Crystal data

C_28_H_30_N_4_O_8_S_2_·C_2_H_6_O
*M* *_r_* = 660.75
Triclinic, 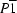

*a* = 7.7619 (15) Å
*b* = 14.473 (3) Å
*c* = 15.810 (3) Å
α = 67.113 (10)°
β = 73.069 (9)°
γ = 78.210 (12)°
*V* = 1556.9 (5) Å^3^
*Z* = 2
Mo *K*α radiation
μ = 0.23 mm^−1^
*T* = 113 (2) K
0.14 × 0.12 × 0.10 mm

#### Data collection

Rigaku Saturn CCD area-detector diffractometer
Absorption correction: multi-scan (*CrystalClear*; Rigaku/MSC, 2005 ▶) *T*_min_ = 0.968, *T*_max_ = 0.977
18692 measured reflections
6824 independent reflections
5694 reflections with *I* > 2σ(*I*)
*R*_int_ = 0.046

#### Refinement

*R*[*F* ^2^ > 2σ(*F* ^2^)] = 0.050
*wR*(*F* ^2^) = 0.115
*S* = 1.07
6824 reflections
433 parameters
H atoms treated by a mixture of independent and constrained refinement
Δρ_max_ = 0.26 e Å^−3^
Δρ_min_ = −0.32 e Å^−3^

### 

Data collection: *CrystalClear* (Rigaku/MSC, 2005 ▶); cell refinement: *CrystalClear*; data reduction: *CrystalStructure* (Rigaku/MSC, 2005 ▶); program(s) used to solve structure: *SHELXS97* (Sheldrick, 2008 ▶); program(s) used to refine structure: *SHELXL97* (Sheldrick, 2008 ▶); molecular graphics: *SHELXTL* (Sheldrick, 2008 ▶); software used to prepare material for publication: *SHELXTL*.

## Supplementary Material

Crystal structure: contains datablocks I, global. DOI: 10.1107/S1600536808023556/hk2504sup1.cif

Structure factors: contains datablocks I. DOI: 10.1107/S1600536808023556/hk2504Isup2.hkl

Additional supplementary materials:  crystallographic information; 3D view; checkCIF report

## Figures and Tables

**Table 1:** Hydrogen-bond geometry (Å, °)

*D*—H⋯*A*	*D*—H	H⋯*A*	*D*⋯*A*	*D*—H⋯*A*
N4—H4*A*⋯O1^i^	0.90 (2)	2.51 (2)	3.403 (2)	173 (2)
N2—H2⋯S1	0.88 (2)	2.69 (2)	3.3527 (19)	133.0 (18)
N2—H2⋯O2	0.88 (2)	1.97 (2)	2.677 (2)	136 (2)
N1—H1⋯O1	0.89 (2)	1.90 (2)	2.621 (2)	137 (2)
N3—H3*A*⋯O9	0.84 (2)	2.23 (2)	2.949 (2)	145 (2)
O9—H9⋯O2	0.80 (3)	2.13 (3)	2.905 (2)	163 (3)

Symmetry code: (i) 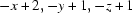.

**Fig. 1.**
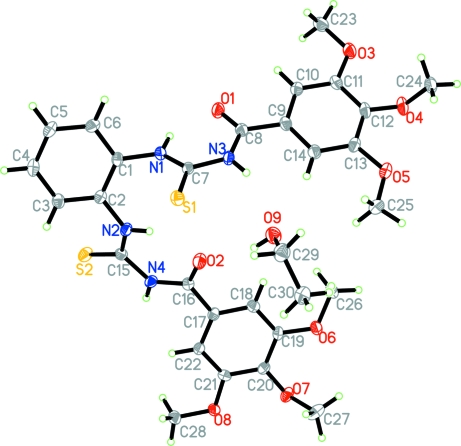
The molecular structure of the title molecule, with the atom-numbering scheme. Displacement ellipsoids are drawn at the 50% probability level.

**Fig. 2.**
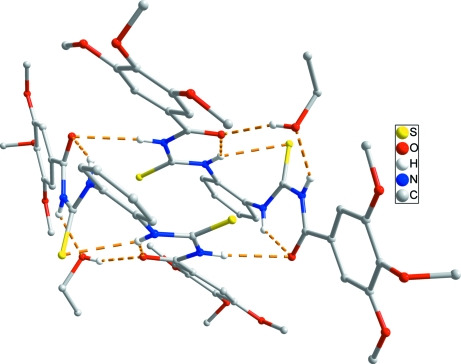
A partial packing diagram of the title compound. Hydrogen bonds are shown as dashed lines.

## supplementary crystallographic information

### Comment

In the molecule of the title compound (Fig. 1) the bond lengths and angles are within normal ranges. Rings A (C1-C6), B (C9-C14) and C (C17-C22) are, of course, planar, and the dihedral angles between them are A/B = 38.50 (6)°, A/C = 5.68 (5)° and B/C = 44.18 (5)°.

The intramolecular N-H···O and N-H···S hydrogen bonds (Table 1) result in the formation of non-planar six-, seven- and eight-membered rings: D (O1/N1/N3/C7/C8/H1), E (O2/N2/N4/C15/C16/H2), F (S1/N1/N2/C1/C2/C7/H2) and G (S1/O2/O9/N3/C7/H2/H3A/H9). Rings D and E adopt flattened-boat [φ = 171.38 (2)°, θ = 109.10 (3)° (for ring D) and φ = -20.28 (3)°, θ = 96.87 (3)° (for ring E)]  conformations, while rings F and G adopt highly twisted conformations having total puckering amplitudes, Q_T_, of 0.160 (3), 0.109 (3), 2.486 (4) and 2.064 (4) Å, respectively (Cremer & Pople, 1975). The two side arms are not twisted in the same way, as evidenced by the torsion angles: C7-N3-C8-O1 [0.1 (3)°], C8-N3-C7-N1 [11.8 (3)°] and C15-N4-C16-O2 [4.6 (3)°], C16-N4-C15-N2 [-11.5 (3)°], as in 1,2-bis(N'-benzoylthioureido)- benzene (Thiam *et al.*,  2008).

In the crystal structure, intermolecular N-H···O hydrogen bonds link the molecules (Fig. 2), in which they may be effective in the stabilization of the structure.

### Experimental

For the preparation of the title compound, ammonium thiocyanate (30 mmol), 3,4,5-trimethoxylbenzoyl chloride (20 mmol), PEG-400 (0.2 mmol) and acetone (50 mL) were placed in a dried round-bottomed flask containing a magnetic stirrer bar and stirred at room temperature for 1 h, then benzene-1,2-diamine (9.5 mmol) was added, and the mixture was stirred for 2 h. The mixture was poured into water (20 ml). The resulting solid was filtered, washed with water, and then dried. Crystals suitable for X-ray analysis were obtained by the recrystallization of the solid residue from a mixture of N,N-dimethyl- formamide/ethanol (1:1) by slow evaporation at room temperature.

### Refinement

H1, H2, H3A, H4A (for NH) and H9 (for OH) atoms were located in difference syntheses and refined isotropically [N-H = 0.84 (2)-0.90 (2) Å and U_iso_(H) = 0.026 (6)-0.036 (7) Å^2^; O-H = 0.80 (3) Å and U_iso_(H) = 0.043 Å^2^]. The remaining H atoms were positioned geometrically, with C-H = 0.95, 0.98 and 0.99 Å for aromatic, methyl and methylene H, respectively, and constrained to ride on their parent atoms with U_iso_(H) = xU_eq_(C), where x = 1.5 for methyl H and x = 1.2 for all other H atoms.

### Crystal data

**Table:**

C_28_H_30_N_4_O_8_S_2_·C_2_H_6_O_1_	*Z* = 2
*M**_r_* = 660.75	*F*_000_ = 696
Triclinic, *P*1	*D*_x_ = 1.409 Mg m^−^^3^
Hall symbol: -P 1	Melting point: 475 K
*a* = 7.7619 (15) Å	Mo *K*α radiation λ = 0.71070 Å
*b* = 14.473 (3) Å	Cell parameters from 4542 reflections
*c* = 15.810 (3) Å	θ = 2.4–27.2º
α = 67.113 (10)º	µ = 0.23 mm^−^^1^
β = 73.069 (9)º	*T* = 113 (2) K
γ = 78.210 (12)º	Block, colorless
*V* = 1556.9 (5) Å^3^	0.14 × 0.12 × 0.10 mm

### Data collection

**Table:**

Rigaku Saturn CCD area-detector diffractometer	6824 independent reflections
Radiation source: rotating anode	5694 reflections with *I* > 2σ(*I*)
Monochromator: confocal	*R*_int_ = 0.046
Detector resolution: 14.63 pixels mm^-1^	θ_max_ = 27.2º
*T* = 113(2) K	θ_min_ = 2.5º
ω scans	*h* = −9→9
Absorption correction: multi-scan(CrystalClear; Rigaku/MSC, 2005)	*k* = −18→18
*T*_min_ = 0.968, *T*_max_ = 0.977	*l* = −20→20
18692 measured reflections	

### Refinement

**Table:**

Refinement on *F*^2^	Hydrogen site location: inferred from neighbouring sites
Least-squares matrix: full	H atoms treated by a mixture of independent and constrained refinement
*R*[*F*^2^ > 2σ(*F*^2^)] = 0.050	*w* = 1/[σ^2^(*F*_o_^2^) + (0.0441*P*)^2^ + 0.4672*P*] where *P* = (*F*_o_^2^ + 2*F*_c_^2^)/3
*wR*(*F*^2^) = 0.115	(Δ/σ)_max_ < 0.001
*S* = 1.07	Δρ_max_ = 0.26 e Å^−^^3^
6824 reflections	Δρ_min_ = −0.32 e Å^−^^3^
433 parameters	Extinction correction: SHELXL97 (Sheldrick, 2008), Fc^*^=kFc[1+0.001xFc^2^λ^3^/sin(2θ)]^-1/4^
Primary atom site location: structure-invariant direct methods	Extinction coefficient: 0.0140 (12)
Secondary atom site location: difference Fourier map	

### Special details

**Table:**

Geometry. All e.s.d.'s (except the e.s.d. in the dihedral angle between two l.s. planes) are estimated using the full covariance matrix. The cell e.s.d.'s are taken into account individually in the estimation of e.s.d.'s in distances, angles and torsion angles; correlations between e.s.d.'s in cell parameters are only used when they are defined by crystal symmetry. An approximate (isotropic) treatment of cell e.s.d.'s is used for estimating e.s.d.'s involving l.s. planes.
Refinement. Refinement of F^2^ against ALL reflections. The weighted R-factor wR and goodness of fit S are based on F^2^, conventional R-factors R are based on F, with F set to zero for negative F^2^. The threshold expression of F^2^ > 2sigma(F^2^) is used only for calculating R-factors(gt) etc. and is not relevant to the choice of reflections for refinement. R-factors based on F^2^ are statistically about twice as large as those based on F, and R- factors based on ALL data will be even larger.

### Fractional atomic coordinates and isotropic or equivalent isotropic displacement parameters (Å^2^)

**Table:**

	*x*	*y*	*z*	*U*_iso_*/*U*_eq_	
S1	0.47072 (7)	0.74462 (4)	0.51329 (3)	0.02065 (14)	
S2	0.93034 (8)	0.44117 (4)	0.34131 (4)	0.02578 (15)	
O1	1.02467 (19)	0.81993 (11)	0.48592 (9)	0.0250 (3)	
O2	0.6648 (2)	0.51201 (10)	0.60921 (10)	0.0274 (4)	
O3	1.2473 (2)	0.92569 (11)	0.70183 (10)	0.0286 (4)	
O4	1.0727 (2)	0.82041 (11)	0.87598 (10)	0.0278 (4)	
O5	0.7886 (2)	0.72356 (11)	0.90656 (10)	0.0287 (4)	
O6	0.7859 (2)	0.27652 (10)	0.93150 (10)	0.0243 (3)	
O7	0.7307 (2)	0.09337 (10)	0.94217 (9)	0.0244 (3)	
O8	0.6871 (2)	0.06352 (10)	0.79539 (9)	0.0229 (3)	
O9	0.5525 (2)	0.62076 (12)	0.74010 (11)	0.0285 (4)	
H9	0.568 (4)	0.582 (2)	0.7125 (19)	0.043*	
N1	0.8159 (2)	0.76073 (12)	0.41701 (11)	0.0177 (4)	
H1	0.923 (3)	0.7772 (17)	0.4133 (16)	0.030 (7)*	
N2	0.7346 (2)	0.57673 (12)	0.42050 (12)	0.0200 (4)	
H2	0.685 (3)	0.5885 (17)	0.4737 (17)	0.028 (6)*	
N3	0.7451 (2)	0.76939 (12)	0.56722 (12)	0.0184 (4)	
H3A	0.673 (3)	0.7516 (17)	0.6201 (17)	0.026 (6)*	
N4	0.7865 (2)	0.41196 (12)	0.52015 (11)	0.0198 (4)	
H4A	0.830 (3)	0.3486 (19)	0.5237 (17)	0.036 (7)*	
C1	0.7839 (3)	0.75352 (14)	0.33508 (13)	0.0175 (4)	
C2	0.7479 (3)	0.66254 (14)	0.33554 (13)	0.0176 (4)	
C3	0.7222 (3)	0.65850 (15)	0.25351 (14)	0.0207 (4)	
H3	0.6911	0.5981	0.2539	0.025*	
C4	0.7419 (3)	0.74278 (16)	0.17098 (14)	0.0238 (5)	
H4	0.7272	0.7392	0.1148	0.029*	
C5	0.7829 (3)	0.83206 (16)	0.17021 (14)	0.0249 (5)	
H5	0.7987	0.8890	0.1134	0.030*	
C6	0.8006 (3)	0.83775 (15)	0.25285 (14)	0.0213 (4)	
H6	0.8242	0.8995	0.2532	0.026*	
C7	0.6875 (3)	0.75825 (13)	0.49576 (13)	0.0167 (4)	
C8	0.9074 (3)	0.79901 (14)	0.56059 (14)	0.0193 (4)	
C9	0.9324 (3)	0.80961 (14)	0.64638 (14)	0.0191 (4)	
C10	1.0706 (3)	0.86638 (14)	0.63139 (14)	0.0199 (4)	
H10	1.1358	0.9001	0.5690	0.024*	
C11	1.1123 (3)	0.87340 (15)	0.70806 (14)	0.0207 (4)	
C12	1.0173 (3)	0.82339 (15)	0.80012 (14)	0.0217 (4)	
C13	0.8750 (3)	0.76923 (15)	0.81379 (13)	0.0215 (4)	
C14	0.8326 (3)	0.76151 (15)	0.73745 (14)	0.0219 (4)	
H14	0.7370	0.7240	0.7471	0.026*	
C15	0.8105 (3)	0.48263 (14)	0.42749 (14)	0.0189 (4)	
C16	0.7225 (3)	0.42760 (15)	0.60517 (14)	0.0197 (4)	
C17	0.7314 (3)	0.33652 (14)	0.69148 (13)	0.0180 (4)	
C18	0.7536 (3)	0.35253 (15)	0.76900 (14)	0.0200 (4)	
H18	0.7662	0.4182	0.7647	0.024*	
C19	0.7570 (3)	0.27097 (15)	0.85280 (13)	0.0193 (4)	
C20	0.7301 (3)	0.17510 (14)	0.86015 (13)	0.0187 (4)	
C21	0.7114 (3)	0.16009 (14)	0.78097 (14)	0.0187 (4)	
C22	0.7134 (3)	0.24057 (14)	0.69593 (13)	0.0172 (4)	
H22	0.7026	0.2304	0.6419	0.021*	
C23	1.3530 (3)	0.97237 (17)	0.60921 (16)	0.0301 (5)	
H23A	1.4118	0.9209	0.5812	0.045*	
H23B	1.4455	1.0069	0.6127	0.045*	
H23C	1.2744	1.0214	0.5701	0.045*	
C24	0.9843 (3)	0.89960 (16)	0.91201 (15)	0.0274 (5)	
H24A	1.0110	0.9651	0.8628	0.041*	
H24B	1.0285	0.8918	0.9667	0.041*	
H24C	0.8532	0.8958	0.9311	0.041*	
C25	0.6375 (3)	0.67215 (18)	0.92291 (16)	0.0349 (6)	
H25A	0.5474	0.7192	0.8903	0.052*	
H25B	0.5834	0.6455	0.9909	0.052*	
H25C	0.6777	0.6164	0.8988	0.052*	
C26	0.8306 (3)	0.37088 (16)	0.92470 (15)	0.0277 (5)	
H26A	0.9337	0.3926	0.8709	0.042*	
H26B	0.8624	0.3629	0.9829	0.042*	
H26C	0.7262	0.4217	0.9156	0.042*	
C27	0.5853 (3)	0.09950 (17)	1.02149 (16)	0.0348 (6)	
H27A	0.6219	0.1337	1.0552	0.052*	
H27B	0.5573	0.0314	1.0643	0.052*	
H27C	0.4777	0.1376	0.9989	0.052*	
C28	0.6740 (3)	0.04397 (15)	0.71577 (14)	0.0249 (5)	
H28A	0.5684	0.0849	0.6929	0.037*	
H28B	0.6608	−0.0276	0.7343	0.037*	
H28C	0.7839	0.0613	0.6654	0.037*	
C29	0.3609 (3)	0.63770 (18)	0.77554 (16)	0.0312 (5)	
H29A	0.2987	0.6509	0.7249	0.037*	
H29B	0.3343	0.6983	0.7937	0.037*	
C30	0.2880 (4)	0.5489 (2)	0.85986 (17)	0.0428 (7)	
H30A	0.3103	0.4893	0.8416	0.064*	
H30B	0.1575	0.5639	0.8824	0.064*	
H30C	0.3489	0.5358	0.9103	0.064*	

### Atomic displacement parameters (Å^2^)

**Table:**

	*U*^11^	*U*^22^	*U*^33^	*U*^12^	*U*^13^	*U*^23^
S1	0.0181 (3)	0.0241 (3)	0.0211 (3)	−0.00133 (19)	−0.0054 (2)	−0.0093 (2)
S2	0.0320 (3)	0.0201 (3)	0.0198 (3)	0.0011 (2)	−0.0011 (2)	−0.0068 (2)
O1	0.0240 (8)	0.0343 (8)	0.0179 (7)	−0.0081 (6)	−0.0015 (6)	−0.0103 (6)
O2	0.0436 (10)	0.0171 (7)	0.0200 (8)	0.0031 (6)	−0.0090 (7)	−0.0070 (6)
O3	0.0308 (9)	0.0363 (9)	0.0256 (8)	−0.0101 (7)	−0.0086 (7)	−0.0137 (7)
O4	0.0350 (9)	0.0317 (8)	0.0237 (8)	0.0064 (6)	−0.0154 (7)	−0.0160 (7)
O5	0.0367 (9)	0.0332 (8)	0.0156 (7)	−0.0084 (7)	−0.0045 (6)	−0.0069 (6)
O6	0.0353 (9)	0.0211 (7)	0.0203 (7)	−0.0044 (6)	−0.0106 (6)	−0.0076 (6)
O7	0.0329 (9)	0.0187 (7)	0.0160 (7)	0.0021 (6)	−0.0059 (6)	−0.0024 (6)
O8	0.0356 (9)	0.0149 (7)	0.0202 (7)	−0.0050 (6)	−0.0100 (6)	−0.0046 (6)
O9	0.0278 (9)	0.0318 (9)	0.0286 (9)	−0.0028 (7)	−0.0046 (7)	−0.0149 (7)
N1	0.0187 (9)	0.0202 (8)	0.0159 (8)	−0.0019 (6)	−0.0051 (7)	−0.0073 (7)
N2	0.0280 (10)	0.0165 (8)	0.0142 (8)	−0.0005 (7)	−0.0056 (7)	−0.0045 (7)
N3	0.0199 (9)	0.0227 (9)	0.0134 (8)	−0.0030 (7)	−0.0032 (7)	−0.0074 (7)
N4	0.0268 (10)	0.0137 (8)	0.0165 (8)	−0.0013 (7)	−0.0036 (7)	−0.0041 (7)
C1	0.0152 (10)	0.0219 (10)	0.0155 (9)	0.0005 (7)	−0.0032 (7)	−0.0084 (8)
C2	0.0171 (10)	0.0183 (9)	0.0158 (9)	0.0013 (7)	−0.0035 (8)	−0.0061 (8)
C3	0.0218 (11)	0.0214 (10)	0.0202 (10)	0.0017 (8)	−0.0066 (8)	−0.0094 (8)
C4	0.0252 (11)	0.0293 (11)	0.0184 (10)	0.0045 (8)	−0.0084 (8)	−0.0112 (9)
C5	0.0285 (12)	0.0248 (11)	0.0155 (10)	0.0012 (8)	−0.0048 (9)	−0.0031 (8)
C6	0.0229 (11)	0.0174 (10)	0.0197 (10)	−0.0010 (8)	−0.0031 (8)	−0.0043 (8)
C7	0.0219 (10)	0.0131 (9)	0.0151 (9)	0.0000 (7)	−0.0069 (8)	−0.0039 (7)
C8	0.0206 (10)	0.0176 (9)	0.0205 (10)	−0.0003 (7)	−0.0067 (8)	−0.0071 (8)
C9	0.0209 (10)	0.0194 (10)	0.0195 (10)	0.0019 (8)	−0.0069 (8)	−0.0098 (8)
C10	0.0201 (10)	0.0198 (10)	0.0190 (10)	0.0009 (8)	−0.0057 (8)	−0.0066 (8)
C11	0.0210 (11)	0.0215 (10)	0.0234 (10)	0.0007 (8)	−0.0072 (8)	−0.0117 (8)
C12	0.0263 (11)	0.0229 (10)	0.0208 (10)	0.0051 (8)	−0.0121 (9)	−0.0119 (8)
C13	0.0254 (11)	0.0217 (10)	0.0151 (10)	0.0019 (8)	−0.0055 (8)	−0.0056 (8)
C14	0.0241 (11)	0.0213 (10)	0.0212 (10)	−0.0013 (8)	−0.0063 (8)	−0.0083 (8)
C15	0.0197 (10)	0.0178 (9)	0.0201 (10)	−0.0032 (7)	−0.0058 (8)	−0.0061 (8)
C16	0.0222 (10)	0.0197 (10)	0.0180 (10)	−0.0037 (8)	−0.0055 (8)	−0.0063 (8)
C17	0.0176 (10)	0.0189 (10)	0.0158 (9)	0.0005 (7)	−0.0034 (8)	−0.0057 (8)
C18	0.0217 (10)	0.0182 (10)	0.0211 (10)	−0.0023 (7)	−0.0051 (8)	−0.0077 (8)
C19	0.0195 (10)	0.0240 (10)	0.0150 (9)	−0.0009 (8)	−0.0047 (8)	−0.0077 (8)
C20	0.0199 (10)	0.0173 (9)	0.0159 (9)	−0.0013 (7)	−0.0047 (8)	−0.0027 (8)
C21	0.0176 (10)	0.0161 (9)	0.0233 (10)	−0.0024 (7)	−0.0054 (8)	−0.0071 (8)
C22	0.0175 (10)	0.0187 (9)	0.0149 (9)	−0.0020 (7)	−0.0037 (7)	−0.0051 (8)
C23	0.0306 (13)	0.0312 (12)	0.0327 (12)	−0.0105 (9)	−0.0052 (10)	−0.0137 (10)
C24	0.0357 (13)	0.0284 (11)	0.0226 (11)	−0.0013 (9)	−0.0092 (9)	−0.0129 (9)
C25	0.0463 (15)	0.0343 (13)	0.0222 (11)	−0.0170 (11)	0.0012 (10)	−0.0083 (10)
C26	0.0365 (13)	0.0264 (11)	0.0263 (11)	−0.0053 (9)	−0.0096 (10)	−0.0129 (9)
C27	0.0348 (13)	0.0300 (12)	0.0250 (12)	−0.0045 (10)	0.0006 (10)	0.0008 (10)
C28	0.0355 (13)	0.0191 (10)	0.0235 (11)	−0.0022 (8)	−0.0110 (9)	−0.0086 (9)
C29	0.0277 (12)	0.0373 (13)	0.0277 (12)	−0.0028 (9)	−0.0076 (10)	−0.0100 (10)
C30	0.0557 (17)	0.0482 (16)	0.0263 (13)	−0.0224 (13)	0.0021 (12)	−0.0150 (12)

### Geometric parameters (Å, °)

**Table:**

S1—C7	1.664 (2)	C9—C10	1.393 (3)
S2—C15	1.659 (2)	C9—C14	1.396 (3)
O1—C8	1.234 (2)	C10—C11	1.386 (3)
O2—C16	1.232 (2)	C10—H10	0.9500
O3—C11	1.373 (2)	C11—C12	1.400 (3)
O3—C23	1.425 (3)	C12—C13	1.402 (3)
O4—C12	1.370 (2)	C13—C14	1.389 (3)
O4—C24	1.438 (2)	C14—H14	0.9500
O5—C13	1.375 (2)	C16—C17	1.491 (3)
O5—C25	1.423 (3)	C17—C18	1.395 (3)
O6—C19	1.361 (2)	C17—C22	1.397 (3)
O6—C26	1.433 (2)	C18—C19	1.393 (3)
O7—C20	1.374 (2)	C18—H18	0.9500
O7—C27	1.441 (3)	C19—C20	1.400 (3)
O8—C21	1.370 (2)	C20—C21	1.402 (3)
O8—C28	1.428 (2)	C21—C22	1.393 (3)
O9—C29	1.434 (3)	C22—H22	0.9500
O9—H9	0.80 (3)	C23—H23A	0.9800
N1—C7	1.342 (2)	C23—H23B	0.9800
N1—C1	1.433 (3)	C23—H23C	0.9800
N1—H1	0.89 (2)	C24—H24A	0.9800
N2—C15	1.344 (3)	C24—H24B	0.9800
N2—C2	1.425 (2)	C24—H24C	0.9800
N2—H2	0.88 (2)	C25—H25A	0.9800
N3—C8	1.375 (3)	C25—H25B	0.9800
N3—C7	1.403 (3)	C25—H25C	0.9800
N3—H3A	0.84 (2)	C26—H26A	0.9800
N4—C16	1.381 (3)	C26—H26B	0.9800
N4—C15	1.407 (2)	C26—H26C	0.9800
N4—H4A	0.90 (2)	C27—H27A	0.9800
C1—C6	1.388 (3)	C27—H27B	0.9800
C1—C2	1.398 (3)	C27—H27C	0.9800
C2—C3	1.393 (3)	C28—H28A	0.9800
C3—C4	1.391 (3)	C28—H28B	0.9800
C3—H3	0.9500	C28—H28C	0.9800
C4—C5	1.387 (3)	C29—C30	1.508 (3)
C4—H4	0.9500	C29—H29A	0.9900
C5—C6	1.388 (3)	C29—H29B	0.9900
C5—H5	0.9500	C30—H30A	0.9800
C6—H6	0.9500	C30—H30B	0.9800
C8—C9	1.493 (3)	C30—H30C	0.9800
			
C11—O3—C23	116.05 (16)	C18—C17—C22	121.48 (17)
C12—O4—C24	114.05 (15)	C18—C17—C16	116.36 (18)
C13—O5—C25	116.56 (17)	C22—C17—C16	122.13 (18)
C19—O6—C26	117.41 (16)	C19—C18—C17	119.18 (18)
C20—O7—C27	114.85 (15)	C19—C18—H18	120.4
C21—O8—C28	117.05 (15)	C17—C18—H18	120.4
C29—O9—H9	107.2 (19)	O6—C19—C18	124.44 (18)
C7—N1—C1	124.35 (17)	O6—C19—C20	115.37 (17)
C7—N1—H1	116.3 (15)	C18—C19—C20	120.19 (18)
C1—N1—H1	118.6 (15)	O7—C20—C19	121.59 (18)
C15—N2—C2	125.79 (17)	O7—C20—C21	118.60 (17)
C15—N2—H2	117.3 (15)	C19—C20—C21	119.73 (17)
C2—N2—H2	116.3 (15)	O8—C21—C22	124.37 (18)
C8—N3—C7	127.62 (17)	O8—C21—C20	115.14 (17)
C8—N3—H3A	118.5 (16)	C22—C21—C20	120.47 (18)
C7—N3—H3A	113.8 (16)	C21—C22—C17	118.83 (18)
C16—N4—C15	129.46 (17)	C21—C22—H22	120.6
C16—N4—H4A	116.0 (15)	C17—C22—H22	120.6
C15—N4—H4A	114.3 (15)	O3—C23—H23A	109.5
C6—C1—C2	120.34 (18)	O3—C23—H23B	109.5
C6—C1—N1	118.33 (18)	H23A—C23—H23B	109.5
C2—C1—N1	121.21 (17)	O3—C23—H23C	109.5
C3—C2—C1	119.20 (18)	H23A—C23—H23C	109.5
C3—C2—N2	121.58 (18)	H23B—C23—H23C	109.5
C1—C2—N2	119.21 (17)	O4—C24—H24A	109.5
C4—C3—C2	120.1 (2)	O4—C24—H24B	109.5
C4—C3—H3	120.0	H24A—C24—H24B	109.5
C2—C3—H3	120.0	O4—C24—H24C	109.5
C5—C4—C3	120.44 (19)	H24A—C24—H24C	109.5
C5—C4—H4	119.8	H24B—C24—H24C	109.5
C3—C4—H4	119.8	O5—C25—H25A	109.5
C4—C5—C6	119.65 (19)	O5—C25—H25B	109.5
C4—C5—H5	120.2	H25A—C25—H25B	109.5
C6—C5—H5	120.2	O5—C25—H25C	109.5
C5—C6—C1	120.21 (19)	H25A—C25—H25C	109.5
C5—C6—H6	119.9	H25B—C25—H25C	109.5
C1—C6—H6	119.9	O6—C26—H26A	109.5
N1—C7—N3	115.67 (17)	O6—C26—H26B	109.5
N1—C7—S1	125.33 (15)	H26A—C26—H26B	109.5
N3—C7—S1	118.99 (14)	O6—C26—H26C	109.5
O1—C8—N3	121.57 (18)	H26A—C26—H26C	109.5
O1—C8—C9	121.22 (18)	H26B—C26—H26C	109.5
N3—C8—C9	117.16 (17)	O7—C27—H27A	109.5
C10—C9—C14	120.91 (19)	O7—C27—H27B	109.5
C10—C9—C8	115.91 (17)	H27A—C27—H27B	109.5
C14—C9—C8	123.07 (18)	O7—C27—H27C	109.5
C11—C10—C9	119.59 (18)	H27A—C27—H27C	109.5
C11—C10—H10	120.2	H27B—C27—H27C	109.5
C9—C10—H10	120.2	O8—C28—H28A	109.5
O3—C11—C10	124.62 (18)	O8—C28—H28B	109.5
O3—C11—C12	114.95 (18)	H28A—C28—H28B	109.5
C10—C11—C12	120.41 (19)	O8—C28—H28C	109.5
O4—C12—C11	120.25 (19)	H28A—C28—H28C	109.5
O4—C12—C13	120.29 (18)	H28B—C28—H28C	109.5
C11—C12—C13	119.24 (18)	O9—C29—C30	112.1 (2)
O5—C13—C14	124.23 (19)	O9—C29—H29A	109.2
O5—C13—C12	115.03 (18)	C30—C29—H29A	109.2
C14—C13—C12	120.71 (18)	O9—C29—H29B	109.2
C13—C14—C9	119.08 (19)	C30—C29—H29B	109.2
C13—C14—H14	120.5	H29A—C29—H29B	107.9
C9—C14—H14	120.5	C29—C30—H30A	109.5
N2—C15—N4	114.99 (17)	C29—C30—H30B	109.5
N2—C15—S2	128.09 (15)	H30A—C30—H30B	109.5
N4—C15—S2	116.90 (14)	C29—C30—H30C	109.5
O2—C16—N4	121.94 (18)	H30A—C30—H30C	109.5
O2—C16—C17	122.15 (18)	H30B—C30—H30C	109.5
N4—C16—C17	115.90 (17)		
			
C7—N1—C1—C6	112.1 (2)	C11—C12—C13—O5	−179.33 (17)
C7—N1—C1—C2	−71.9 (2)	O4—C12—C13—C14	−171.96 (17)
C6—C1—C2—C3	−2.4 (3)	C11—C12—C13—C14	2.6 (3)
N1—C1—C2—C3	−178.42 (17)	O5—C13—C14—C9	−178.68 (18)
C6—C1—C2—N2	178.93 (17)	C12—C13—C14—C9	−0.8 (3)
N1—C1—C2—N2	2.9 (3)	C10—C9—C14—C13	−1.2 (3)
C15—N2—C2—C3	46.2 (3)	C8—C9—C14—C13	174.92 (18)
C15—N2—C2—C1	−135.2 (2)	C2—N2—C15—N4	176.38 (18)
C1—C2—C3—C4	3.5 (3)	C2—N2—C15—S2	−2.4 (3)
N2—C2—C3—C4	−177.94 (18)	C16—N4—C15—N2	−11.5 (3)
C2—C3—C4—C5	−1.6 (3)	C16—N4—C15—S2	167.45 (17)
C3—C4—C5—C6	−1.3 (3)	C15—N4—C16—O2	4.6 (3)
C4—C5—C6—C1	2.3 (3)	C15—N4—C16—C17	−174.03 (19)
C2—C1—C6—C5	−0.5 (3)	O2—C16—C17—C18	−28.7 (3)
N1—C1—C6—C5	175.64 (18)	N4—C16—C17—C18	149.99 (18)
C1—N1—C7—N3	−178.21 (16)	O2—C16—C17—C22	149.5 (2)
C1—N1—C7—S1	1.0 (3)	N4—C16—C17—C22	−31.8 (3)
C8—N3—C7—N1	11.8 (3)	C22—C17—C18—C19	−0.3 (3)
C8—N3—C7—S1	−167.47 (15)	C16—C17—C18—C19	177.93 (18)
C7—N3—C8—O1	0.1 (3)	C26—O6—C19—C18	−5.4 (3)
C7—N3—C8—C9	177.21 (17)	C26—O6—C19—C20	174.43 (17)
O1—C8—C9—C10	15.1 (3)	C17—C18—C19—O6	176.90 (17)
N3—C8—C9—C10	−162.03 (17)	C17—C18—C19—C20	−2.9 (3)
O1—C8—C9—C14	−161.21 (19)	C27—O7—C20—C19	65.1 (3)
N3—C8—C9—C14	21.6 (3)	C27—O7—C20—C21	−118.3 (2)
C14—C9—C10—C11	1.4 (3)	O6—C19—C20—O7	0.9 (3)
C8—C9—C10—C11	−174.97 (17)	C18—C19—C20—O7	−179.27 (17)
C23—O3—C11—C10	−2.1 (3)	O6—C19—C20—C21	−175.70 (17)
C23—O3—C11—C12	176.46 (17)	C18—C19—C20—C21	4.1 (3)
C9—C10—C11—O3	178.88 (18)	C28—O8—C21—C22	4.0 (3)
C9—C10—C11—C12	0.4 (3)	C28—O8—C21—C20	−177.79 (17)
C24—O4—C12—C11	93.5 (2)	O7—C20—C21—O8	2.8 (3)
C24—O4—C12—C13	−92.0 (2)	C19—C20—C21—O8	179.51 (17)
O3—C11—C12—O4	−6.4 (3)	O7—C20—C21—C22	−178.87 (17)
C10—C11—C12—O4	172.17 (17)	C19—C20—C21—C22	−2.2 (3)
O3—C11—C12—C13	178.98 (17)	O8—C21—C22—C17	177.19 (17)
C10—C11—C12—C13	−2.4 (3)	C20—C21—C22—C17	−1.0 (3)
C25—O5—C13—C14	−5.1 (3)	C18—C17—C22—C21	2.2 (3)
C25—O5—C13—C12	176.94 (18)	C16—C17—C22—C21	−175.89 (18)
O4—C12—C13—O5	6.1 (3)		

### Hydrogen-bond geometry (Å, °)

**Table:**

*D*—H···*A*	*D*—H	H···*A*	*D*···*A*	*D*—H···*A*
N4—H4A···O1^i^	0.90 (2)	2.51 (2)	3.403 (2)	173 (2)
N2—H2···S1	0.88 (2)	2.69 (2)	3.3527 (19)	133.0 (18)
N2—H2···O2	0.88 (2)	1.97 (2)	2.677 (2)	136 (2)
N1—H1···O1	0.89 (2)	1.90 (2)	2.621 (2)	137 (2)
N3—H3A···O9	0.84 (2)	2.23 (2)	2.949 (2)	145 (2)
O9—H9···O2	0.80 (3)	2.13 (3)	2.905 (2)	163 (3)

Symmetry codes: (i) −*x*+2, −*y*+1, −*z*+1.

## References

[bb1] Cremer, D. & Pople, J. A. (1975). *J. Am. Chem. Soc.* **97**, 1354–1358.

[bb2] Rigaku/MSC. (2005). *CrystalClear* and *CrystalStructure* Rigaku/MSC, The Woodlands, Texas, USA.

[bb3] Sheldrick, G. M. (2008). *Acta Cryst.* A**64**, 112–122. 10.1107/S0108767307043930 18156677

[bb4] Thiam, E. I., Diop, M., Gaye, M., Sall, A. S. & Barry, A. H. (2008). *Acta Cryst.* E**64**, o776. 10.1107/S1600536808008374 PMC2961338 21202269

